# The Relation of the Nervous System to Tuberculosis

**Published:** 1912-09

**Authors:** J. Cheston King

**Affiliations:** Atlanta, Ga.


### THE RELATION OF THE NERVOUS SYSTEM TO TUBERCULOSIS.

By J. Cheston King, A. B., M. D., Atlanta, Ga.

Mr. President and Gentlemen:

My subject is one of the every-day observations with which the specialist and general practitioner is called upon to treat.

All I can do for the present is to lay down some general principles and plain observations which will guide us in prognosis and treatment. Whatever theoretical notions the physicians have had as to the nature or treatment of disease, when brought face to face with the sick and suffering, he is compelled to submit every question to the crucial test of actual observation. His duty lies in giving an exact and scientific character to his observations and to investigate the phenomena of disease with that concentration which is necessary in every physical inquiry, and with all those aids which are afforded in increasing perfection by modern science. He cannot afford to have a certain system to satisfy, or certain dogmatic opinions to enforce. He should be untrammelled by any exclusive theory in pathology or therapeutics, but should investigate disease in every possible way and by all the appliances of medical science.

And while I can only present to you that which is old, yet the old is not to be underrated.

Ribot has well said: "The new perishes, the old endures." The saying is a partial truth. The seed that is old produces a new plant. The rising sun is old enough, but each sunrise is a new event. A quarter of a century ago, comparatively little was known as regards the nervous system. We had no conception of modern means of investigation and research which are applied daily to the discovery and explanation of physiological and pathological phenomena.

The labors of the Anatomist, Physiologist and Pathologist are at the present moment establishing an intimate relationship between the minute anatomy of the nervous system and the lungs.

Careful observers of this day are bringing many of the results obtained by vivisection into harmony with pathological data.

First, what do we understand by the nervous system? It includes the neuroglia which supports and separates the nerve element, the blood vessels which penetrate and permeate the centres and the membranes which enclose and protect them. Now, disease in any of the nerve elements transform the said elements into fibres, which contract in a cicatrical change, or they undergo fatty degeneration and caseation. This caseation occurs in several discrete spots, perhaps because the vessels of the neoplasm become shrunken and closed. These areas coalesce and so often they run into the general uniform caseation, so characteristic of the tuberculous mass.

So we can readily see that lesions in one part of the body may reflexly through the nervous system originate morbid changes in a distant part.

Thus chilling of one hand will reduce the temperature of the other; cutaneous irritation transmit its impulses to the base of the brain.

In fact, nervous impairments create organic disease. Thought, grief, joy, and other mental perturbations, often manifest themselves in blanching of the skin, polyuria, glycosuria, vomiting and syncope.

Now, any alteration of the normal trophic mechanism often produces a toxic condition in the body. We know that milk secreted by mothers after a severe fright may be highly toxic.

The brain receives its impulses through the nervous mechanism and each part of the brain has its special function and to a certain extent dependent on the other. One part is essential to vital processes, hence its destruction causes death; another part presides over the movements of the body, hence often paralysis of motion; a third part enables the mind to appreciate touch, temperature and pain; a fourth organ presides over sight; and in the same way this pilot of the nervous machinery governs the amount of oxygen taken into the system.

So we can readily comprehend how the various emotions consequent upon mental strain produce glycosuria and diabetes mellitus, which are potent factors in the development of pulmonary tuberculosis.

Physical condition is not independent of mental action and there is no pathological condition of the individual in which both mind and body are not affected, but in some instances the mental symptoms come into prominence, in others the physical.

In the present age, the means of physical diagnosis and of careful clinical study have increased with unexampled rapidity and no bounds can be set to the study of the intricate nervous system and that dreaded of all diseases—tuberculosis.

Half a century ago there was no conception of these means of modern investigation and research which are applied daily to the discovery and explanation of physiological and pathological phenomena.

To the interpretation of sound heard within the body Leannec and a host of subsequent observers brought precise acoustical observation and experimnts and showed us how to map out the condition of internal parts, the action of which we hear.

To Desormeaux and Cruise belong the honor of having laid open to us by optical instruments every organ of the body, before inscrutable. So what was once hidden phenomena is now practical demonstration. Helmhatz and others have disclosed the physical contrivances of the eye.

The skillful apparatus of Maxey registers every phenomena of the pulse and heart. We thereby not only fathom the secrets of the circulating apparatus, but of nerve action and nerve lesion behind and beyond.

In every practitioner's hand the microscope and the test-tube answer in a moment questions of the greatest moment.

The explanations of the nervous system by physical agencies, through the labors of Duchenne, Oppenheim, Graves and others, may be cited only as advances at our command to connect accurately the relationship one disease bears to another.

Dr. John Hunter, in his experiments and researches, established the close relationship of the cerebro, spinal and sympathetic nervous system to the processes of respiration, circulation, nutrition and temperature. All living matter undergoes cyclical changes. All living matter proceeds from pre-existing living matter and if it is the offshoot of a disarranged bundle of nerve fibres, the human mechanism is impaired and that part of the machinery which is most vulnerable is attacked.

So, seeing how closely interwoven are the different parts of the human mechanism, we are called upon as physicians to study the complicated apparatus of the nervous system and its control over the human system; and the complicated muscular apparatus obeying the volitions of the mind through the nervous system and accomplishing varying mechanical actions by which matter is molded and forces controlled and directed according to the interior ideal creations of the intellectual faculties. The office of the digestive, circulatory and respiratory system is to prepare materials which will readily enter into chemical changes and thus generate the forces by which, under the guidance of the nervous mechanism, the locomotive apparatus of the body may be moved and the barriers and obstacles in nature overcome, and the forces of nature controlled and directed so as to accomplish definite results.

It is often the case that a patient comes to us with so much damage to the nervous system, arterial disease so pronounced, with the motor functions involved, thereby constantly losing the proper amount of oxygenation, then tubercular conditions follow.

It is not often, now, that the initial disease is undetected in consequence of the ignorance of the doctor who is consulted, but it happens sometimes because he does not make a sufficiently careful search to discover how the symptoms of one disease influence the significance of another, and of the need for comparing them, of weighing them alone and together, in order to discern the resultant of their several forces, whether the radical cure of one would eliminate the other.

Successful treatment only follows a careful discernment of disease. By it we arrive at the case, which is the foundation of our school of medicine. Without these proper lines of demarkation, close kinship of the nervous system to the most fatal of all diseases, tuberculosis, treatment is as a "Thistledown without seed."

It is by research and comparison that we attain a knowledge that is persistent and not that which is merely received, and such knowledge gives us an influence into every branch of the complex activity of practical life. We must remember that the life of the muscular system depends on the nerves. Slow atrophy of the muscle follows slow degeneration of the nerve, a relation we may call functional vitality.

The loss of nutritional vigor of the nervous system leaves a structure made to resist the slightest influence tubercular infection. If the nerves slowly degenerate, so do the muscles; if rapidly, from descending irritation, the muscles undergo speedy complete degeneration. We can go back into the distant past until we are lost in the blue mists, which shroud alike the hills of Greece and the deserts of Arabia, or to the time when the world learned its wisdom from the land where now the symbols of man's thought lie deep beneath the desert sand, or stand silent in the cold moonlight of a long dead past, and find sirens singing praises of the sages of medicine studying the intimate relationship of life, and the kinship of one disease to another.

But to the praise of our present generation of physicions, let it be said, that their march in research has been steady. They appreciate the fact "that knowledge which a man can use is the only real knowledge which has life and growth in it and converts itself into practical power; the rest hangs like dust about the brain or dies like raindrops off the stone."

